# Autism Associated Gene, *ENGRAILED2*, and Flanking Gene Levels Are Altered in Post-Mortem Cerebellum

**DOI:** 10.1371/journal.pone.0087208

**Published:** 2014-02-10

**Authors:** Jiyeon Choi, Myka R. Ababon, Mai Soliman, Yong Lin, Linda M. Brzustowicz, Paul G. Matteson, James H. Millonig

**Affiliations:** 1 Center for Advanced Biotechnology and Medicine, Piscataway, New Jersey, United States of America; 2 Cancer Institute of New Jersey, Piscataway, New Jersey, United States of America; 3 Department of Neuroscience and Cell Biology, Robert Wood Johnson Medical School, Piscataway, New Jersey, United States of America; 4 Department of Genetics, Rutgers University, Piscataway, New Jersey, United States of America; Simon Fraser University, Canada

## Abstract

**Background:**

Previous genetic studies demonstrated association between the transcription factor *ENGRAILED2* (*EN2*) and Autism Spectrum Disorder (ASD). Subsequent molecular analysis determined that the *EN2* ASD-associated haplotype (*rs1861972*-*rs1861973* A-C) functions as a transcriptional activator to increase gene expression. *EN2* is flanked by 5 genes, *SEROTONIN RECEPTOR5A (HTR5A), INSULIN INDUCED GENE1 (INSIG1)*, *CANOPY1 HOMOLOG (CNPY1), RNA BINDING MOTIF PROTEIN33 (RBM33)*, and *SONIC HEDGEHOG (SHH)*. These flanking genes are co-expressed with *EN2* during development and coordinate similar developmental processes. To investigate if mRNA levels for these genes are altered in individuals with autism, post-mortem analysis was performed.

**Methods:**

qRT-PCR quantified mRNA levels for *EN2* and the 5 flanking genes in 78 post-mortem cerebellar samples. mRNA levels were correlated with both affection status and *rs1861972-rs1861973* genotype. Molecular analysis investigated whether *EN2* regulates flanking gene expression.

**Results:**

*EN2* levels are increased in affected A-C/G-T individuals (p = .0077). Affected individuals also display a significant increase in *SHH* and a decrease in *INSIG1* levels. *Rs1861972*-*rs1861973* genotype is correlated with significant increases for *SHH* (A-C/G-T) and *CNPY1* (G-T/G-T) levels. Human cell line over-expression and knock-down as well as mouse knock-out analysis are consistent with *EN2* and *SHH* being co-regulated, which provides a possible mechanism for increased *SHH* post-mortem levels.

**Conclusions:**

*EN2* levels are increased in affected individuals with an A-C/G-T genotype, supporting *EN2* as an ASD susceptibility gene. *SHH*, *CNPY1*, and *INSIG1* levels are also significantly altered depending upon affection status or *rs1861972*-*rs1861973* genotype. Increased *EN2* levels likely contribute to elevated *SHH* expression observed in the post-mortem samples

## Introduction

Autism Spectrum Disorder (ASD) causes deficits in language and social skills along with increased repetitive interests and behaviors. ASD includes autism, Asperger Syndrome (AS), and Pervasive Developmental Disorder-Not Otherwise Specified (PDD-NOS). Individuals with autism display the most severe phenotypes while individuals with AS and PDD-NOS typically exhibit milder symptoms.

Genetic studies have implicated the homeobox transcription factor *ENGRAILED2 (EN2)* as an ASD susceptibility gene. We determined previously that the *EN2* intronic *rs1861972*-*rs1861973* haplotype is associated with ASD, with the A-C haplotype being over-transmitted to affected individuals while the other common haplotype (G-T) is over-represented in unaffected siblings [1], [2]. Six other groups have also reported *EN2* association with ASD [3]–[8].

Our molecular genetic and biochemical studies demonstrated that the ASD-associated A-C haplotype functions as a transcriptional activator [9]. Luciferase assays determined that ASD-associated A-C haplotype is both sufficient and necessary for this activator function. A protein complex specifically binds to the A-C haplotype. These proteins were partially purified and two transcription factors, CUX1 and NFIB, were identified. Subsequent electromobility shift assays (EMSAs), over-expression and knock-down studies indicate that CUX1 and NFIB bind the A-C haplotype at the same time and both proteins are required to mediate the activator function. These data demonstrate that the ASD-associated A-C haplotype is a functional *cis*-regulatory element, and suggest that increased *EN2* levels contribute to ASD risk [9]. Consistent with this possibility, recent study demonstrated that human *EN2* is epigenetically regulated and increased levels are observed in individuals with autism but these results were not correlated with the *rs1861972*-*rs1861973* haplotype [10].

*EN2* also regulates developmental processes relevant to ASD. Numerous groups have demonstrated that *En2* is essential for the topographic mapping of axons in both the tectum and cerebellum [11]–[15]. A recent study suggests that *En2* is also important for maintaining excitatory/inhibitory balance [16]. Finally several groups have demonstrated that *En2* is required for the development of ventral mid-hindbrain neurotransmitter systems [17]–[23].

*EN2* maps to a gene poor region of the genome but the five genes within 1Mb of *EN2* are co-expressed during brain development and perform similar biological functions. These genes are: *SONIC HEDGEHOG* (*SHH*), *INSULIN INDUCED GENE1* (*INSIG1*), *CANOPY1 HOMOLOG* (*CNPY1*), *SEROTONIN RECEPTOR5A* (*HTR5A*), and *RNA BINDING MOTIF PROTEIN33* (*RBM33*).

*SHH* is a morphogen that coordinates many aspects of CNS development including patterning, proliferation, and connectivity. *Shh* and *En2* are co-expressed in the embryonic mid-hindbrain as well as the post-natal and adult cerebellum. During embryogenesis Shh and En2 play important roles in the development of ventral mid-hindbrain neurotransmitter systems [24]–[26]. In the cerebellum Shh promotes granule cell neurogenesis while En2 inhibits proliferation [27], [28].

*INSIG1* regulates cholesterol biosynthesis, and cholesterol modification is needed for Shh activity [29], [30]. *Insig1*, *Shh*, and *En2* are co-expressed in the embryonic mid-hindbrain as well as the adult cerebellum. Interestingly, Smith-Lemli-Opitz Syndrome (SLOS) is a mono-genic disorder with defects in cholesterol biosynthesis. 50-86% of individuals with SLOS are diagnosed with ASD, suggesting an involvement of cholesterol metabolism in the disorder [31].

*CNPY1* is a positive regulator of Fgf signaling and Cnpy1 induces *En2* expression in the developing mid-hindbrain [32], [33]. The Fgf pathway is necessary for neurogenesis, survival, and connectivity, all of which have been implicated in ASD [34]. *Cnpy1* and *En2* are also co-expressed in the adult cerebellum.

*HTR5A* is a 5HT receptor and abnormalities in the serotonin pathway have been consistently implicated in ASD [35]. *Htr5a* is co-expressed with *En2* throughout development and in the adult cerebellum. Finally, *RBM33* contains homology to a predicted RNA binding domain. RNA binding proteins regulate multiple aspects of RNA processing including splicing, translation, and in neurons transport mRNAs to the synapse for local translation [36]. *Rbm33* expression has not been analyzed developmentally but is detected with the other flanking genes in the adult cerebellum. Together these results indicate En2 and the flanking genes regulate similar developmental processes and are co-expressed during development, suggesting they may be co-regulated.

Given the ASD-associated A–C haplotype functions as a transcriptional activator in cultured mouse neurons and human cell lines [9], the next important question is whether the A–C haplotype and affection status are correlated with altered *EN2* expression in individuals with autism. To investigate this possibility, post-mortem analysis was performed. We asked if affection status and *rs1861972-rs1861973* genotype are correlated with *EN2* levels. Because the flanking genes regulate ASD relevant developmental functions and are co-expressed with *EN2*, their levels were also examined and correlated with affection status and *rs1861972-rs1861973* genotype.

## Results

### *EN2* levels are elevated in post-mortem cerebellum

To investigate if *EN2* levels are altered in individuals affected with autism, 90 age and sex matched cerebellar post-mortem samples (35 autism and 55 control) were obtained from NICHD Brain and Tissue Bank for Developmental Disorders, and Harvard Brain Tissue Resource Center (Tables S1 and S2 in File S1). The adult cerebellum was chosen as a structure to analyze because *EN2* and all the flanking genes are expressed in the cerebellum, and our previous molecular analysis demonstrated that the ASD-associated A–C haplotype is functional in mouse cerebellar neurons. To assess genotype effects on *EN2* levels, genomic DNA was isolated and each sample was genotyped for *rs1861972* and *rs1861973*. The three genotypes (A-C/A-C, A-C/G-T, and G-T/G-T) were equally distributed between autism and control groups (Table S3 in File S1). To measure *EN2* mRNA levels, total RNA was also isolated and quality was assessed using RNA integrity number (RIN). 78 samples (29 autism and 49 control) with RIN values greater than 3 were used for the final analysis (Table S1 in File S1). cDNA was then generated and Taqman qRT-PCR was performed in triplicate for each sample and normalized to *GAPDH*.

Post-mortem analysis results can be significantly affected by covariates including: affection, genotype, age, sex, post-mortem interval (PMI), RNA integrity number (RIN), as well as interaction between affection, genotype, and sex (affection*genotype, affection*sex, genotype*sex, and affection*genotype*sex). To take this into account all statistical analysis was adjusted for these covariates. For each gene expression level, insignificant covariates were removed and then a statistical model was established to correct for any significant covariates (see methods for details). Prescription drug use and comorbidity information is also available for a subset of affected individuals but matching information is mostly absent for control individuals rendering covariate analysis impossible. If no drug use or comorbidity in control group is assumed, autism affection status was positively correlated with both prescription drug use and comorbid disorders like epilepsy (P<.0001).

While *EN2* was expressed at higher levels in the post-mortem samples from individuals with autism (Figure S1 in File S1), analysis of covariance for *EN2* levels demonstrated a significant interaction between affection and genotype (p = 0.0006) (Table S4 in File S1). This result indicates the effect of the *rs1861972*-*rs1861973* haplotype is significantly different between the autism and control groups. Based on this finding, *rs1861972-rs1861973* genotype effects on *EN2* levels were compared separately for the affected and control groups.

**Figure 1:**
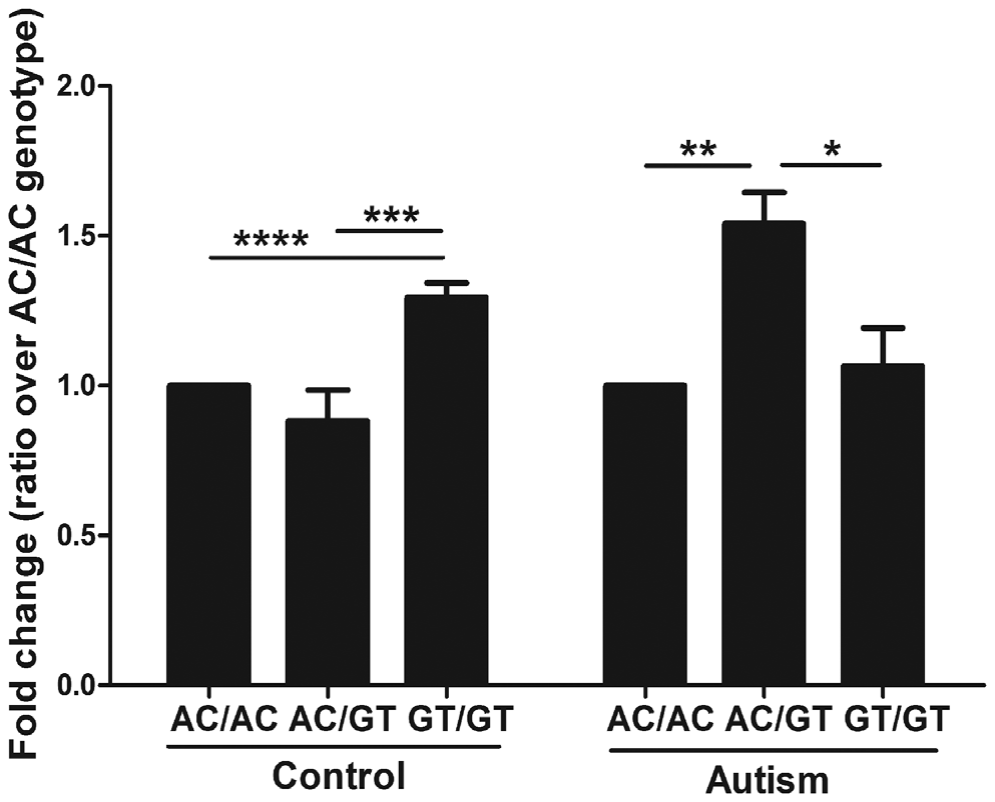
*EN2* levels are elevated in individuals with autism and an A-C/G-T genotype. *EN2* mRNA levels were measured in 29 autism and 49 control cerebellar samples using Taqman qRT-PCR. Based on the interaction between genotype and affection status, *EN2* levels were compared between genotypes (A-C/A-C, A-C/G-T, G-T/G-T) in control and autism groups separately. In each group *EN2* levels are normalized to 1 for A-C/A-C genotype and presented as fold change for the other genotypes (*EN2* levels are similar between control A-C/A-C and autism A-C/A-C group (<0.5% difference). Fold difference was calculated based on ΔΔCt values. Type 3 tests of fixed effects, *P<.05, **P<.01, ***P<.001, ****P<.0001. AC/AC – individuals homozygous for the *rs1861972-rs1861973* A-C haplotype, GT/GT – individuals homozygous for the G-T haplotype, AC/GT – individuals heterozygous for the A-C/G-T haplotype.

For individuals with autism, *EN2* levels exhibited significant differences among three genotypes (p = 0.0225)(Fig 1). Affected A-C/G-T heterozygotes displayed significantly higher *EN2* levels than two other genotypes (54% higher than the A-C/A-C, p = 0.0077 and 45% higher than the G-T/G-T, p = 0.0243) (Table 1 and Fig 1). For control individuals, *EN2* levels also exhibited significant differences among three genotypes (p<0.0001) (data not shown). However, G-T/G-T homozygotes displayed the highest *EN2* levels in control individuals (29% higher than the A-C/A-C, p<.0001, and 47% higher than the A-C/G-T, p = 0.0001) while A-C/G-T is the lowest (Table 1 and Fig 1). In other words, individuals with autism display 74% higher *EN2* levels than controls within A-C/G-T group (p = 0.0005) (Table 1 and Figure S2 in File S1). Although the effect of RIN is already taken into account in our statistical model, we performed the same analyses using samples of RIN>5(N = 59). The results displayed a similar pattern of changes albeit with slightly compromised significance due to the decreased number of samples.

**Table 1:** *EN2* levels comparisons between affection and genotype groups.

Genotype	Affection	Least squared mean^a^	Standard errors^b^	P-value^c^	Fold change^d^	Standard errors^e^
AC/AC	Autism vs. Control	0.007615	0.1462	0.9586	0.9947	0.1015
AC/GT		−0.7963	0.2188	**0.0005**	1.7366	0.2678
GT/GT		0.2897	0.1599	0.0744	0.8180	0.0915
Affection	Genotype	Least squared mean^a^	Standard errors^b^	P-value^c^	Fold change^d^	Standard errors^e^
Autism	AC/AC vs. AC/GT	0.6236	0.2273	**0.0077**	0.6490	0.1041
	AC/AC vs. GT/GT	0.09040	0.1940	0.6426	0.9393	0.1280
	AC/GT vs. GT/GT	−0.5332	0.2315	**0.0243**	1.4471	0.2366
Control	AC/AC vs. AC/GT	−0.1803	0.1300	0.1699	1.1331	0.1027
	AC/AC vs. GT/GT	0.3725	0.08992	**<.0001**	0.7724	0.0483
	AC/GT vs. GT/GT	0.5527	0.1373	**0.0001**	0.6817	0.0653

a Least squared means were calculated from ΔΔCt values (ΔCt^autism^–ΔCt^control^ or ΔCt^1st genotype^–ΔCt^2nd genotype^) after adjusting for significant covariates.

b Standard error for least squared mean.

c Type 3 tests of fixed effects were performed considering all the significant covariates. Significant values (5% cut-off) are in bold.

d Fold change of each first group versus the second is calculated as 2^−(least squared mean)^.

e Standard error of fold change.

These data demonstrate that *EN2* levels are elevated in A-C/G-T affected individuals. In addition, different *rs1861972-rs1861973* genotypes are correlated with increased *EN2* levels in affected (A-C/G-T) and control (G-T/G-T) individuals.

### Mediators of A-C haplotype function are not increased in post-mortem cerebellum

Since the transcription factors, CUX1 and NFIB bind the A-C haplotype and mediate its function, their mRNA levels were also measured in the post-mortem samples. No significant interaction between affection status and genotype was observed for either gene. In addition, no significant difference in mRNA level was observed between affected and control groups or between the three genotype groups for either gene (Figure S3, Tables S5 and S6 in File S1). These data determined *CUX1* and *NFIB* transcript levels are not altered by affection status or *rs1861972-rs1861973* genotype in post-mortem cerebellum.

### Post-mortem expression analysis for five flanking genes of *EN2*

**Figure 2:**
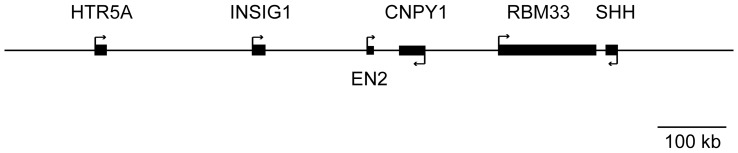
Genomic map of *EN2* and five flanking genes. Genomic region encompassing 500 kb upstream and 500 kb downstream of *EN2* gene is illustrated and drawn to scale. Solid boxes represent each gene and arrows indicate the transcribed DNA strand. Gene symbols for each gene are shown.

Five genes flank *EN2* in a 1Mb region: *HTR5A* and *INSIG1* 5′ of *EN2* while *CNPY1*, *RBM33*, and *SHH* map 3′ of *EN2* (Fig 2). Animal studies indicate that *En2* and these 5 flanking genes are co-expressed (www.brain-map.org; genome.ucsc.edu/cgi-bin/hgVisiGene) and coordinate similar neurodevelopmental processes. These data led us to hypothesize that levels of the flanking genes may also be altered in the cerebellar post-mortem samples.

To examine this possibility, normalized mRNA levels were measured in the same 29 affected and 49 control individuals by Taqman qRT-PCR. Analysis of covariance resulted in slightly different final models for each gene (Tables S7-9 in File S1). Unlike *EN2*, no significant interaction between affection and genotype was observed for any of the five genes. Thus *EN2* is the only gene in the 1Mb area, whose expression levels display an interaction between affection status and *rs1861972-rs1861973* genotype. Based on this finding, comparisons for the five flanking genes were made separately for affection status and genotype.

### *SHH* and *INSIG1* levels are altered in autism

**Figure 3:**
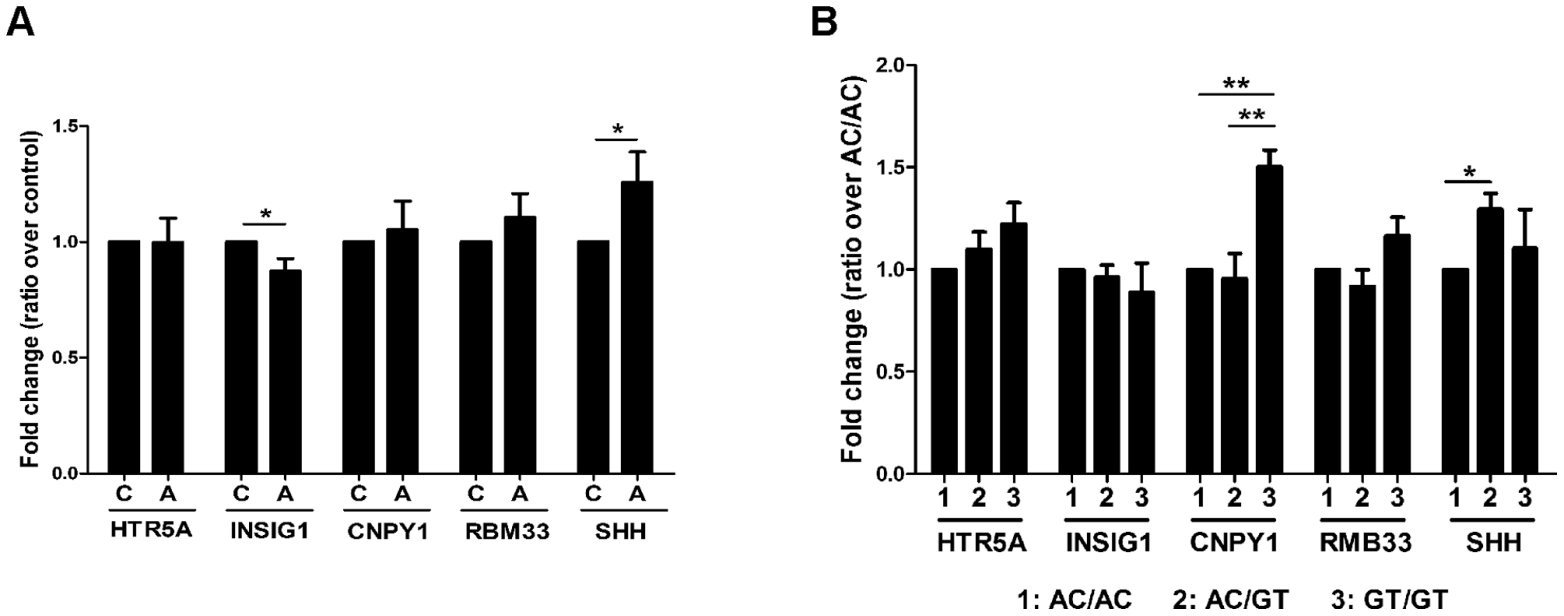
*SHH,INSIG1*, and *CNPY1* levels are correlated with affection status or *rs1861972-rs1861973* genotype. mRNA levels of the five flanking genes were measured using Taqman qRT-PCR. No interaction between affection status and genotype was observed, so comparisions between autism and control group were made regardless of genotype (A) and between the three genotypes regardless of affection status (B). (A) For each gene control levels are normalized to 1,and transcript levels are presented as fold change in autism using ΔΔCt values. Type 3 tests of fixed effects, *p<0.05. Note that *SHH* and *INSIG1* levels are significantly altered in affected individuals. C – control, A – autism group. (B) Each transcript level is normalized to 1 for the genotype with the lowest quantity (AC/AC for *HTR5A*; GT/GT for *INSIG1*; AC/GT for *CNPY1*; AC/GT for *RBM33*; AC/AC for *SHH*). Levels for other genotypes are presented as fold change using ΔΔCt values. Type 3 tests of fixed effects, *p<0.05, **p<0.01. *CNPY1* and *SHH* levels are correlated with *rs1861972-rs1861973* genotype.

After adjusting for covariates, *SHH* levels exhibited a significant increase in the affected group compared to control (26% increase, p = 0.0327) (Fig 3A, Table S10 in File S1). *INSIG1*, on the other hand, displayed decreased levels in affected individuals compared to control (13% decrease, p = 0.0352) (Fig 3A, Table S11 in File S1). None of the other genes displayed a significant difference. These data demonstrate *SHH* and *INSIG1* levels are altered in these individuals with autism (Table 2 for summary).

**Table 2:** Summary of flanking gene results.

	Post-mortem	*EN2* over-expression	*EN2* knock-down	*CUX1-NFIB* knock-down	*En2* knock-out
*SHH*	↑^a^	↑	↓	↓	↓
*CNPY1*	↑^b^	NS^d^	↓	↓	↓
*INSIG1*	↓^c^	↑	NS	↓	NS
*RBM33*	NS	NS	↓	NS	ND^e^
*HTR5A*	NS	↑	↓	↓	ND

a increased in A-C/G-T individuals and affected individuals.

b increased in G-T/G-T individuals.

c decreased in affected individuals.

d NS-not significant.

e ND-not determined.

### *SHH* and *CNPY1* levels are correlated with *rs1861972-rs1861973* genotypes

Differences between the three genotypes were then compared regardless of affection status. *SHH* levels displayed a significant increase in A-C/G-T heterozygotes compared to A-C/A-C homozygotes (30% increase, p = 0.0297) (Fig 3B, Table S10 in File S1). *CNPY1* exhibited increased levels in G-T/G-T homozygotes compared to A-C/A-C and A-C/G-T individuals (50%, p = 0.0037 and 57% increase, p = 0.0013, respectively) (Fig 3B, Table S12 in File S1). None of the other genes displayed a significant difference. These data demonstrate that the *rs1861972-rs1861973* genotype is correlated with altered *SHH* and *CNPY1* levels (Table 2 for summary).

### *EN2* and *SHH* co-regulation

**Figure 4:**
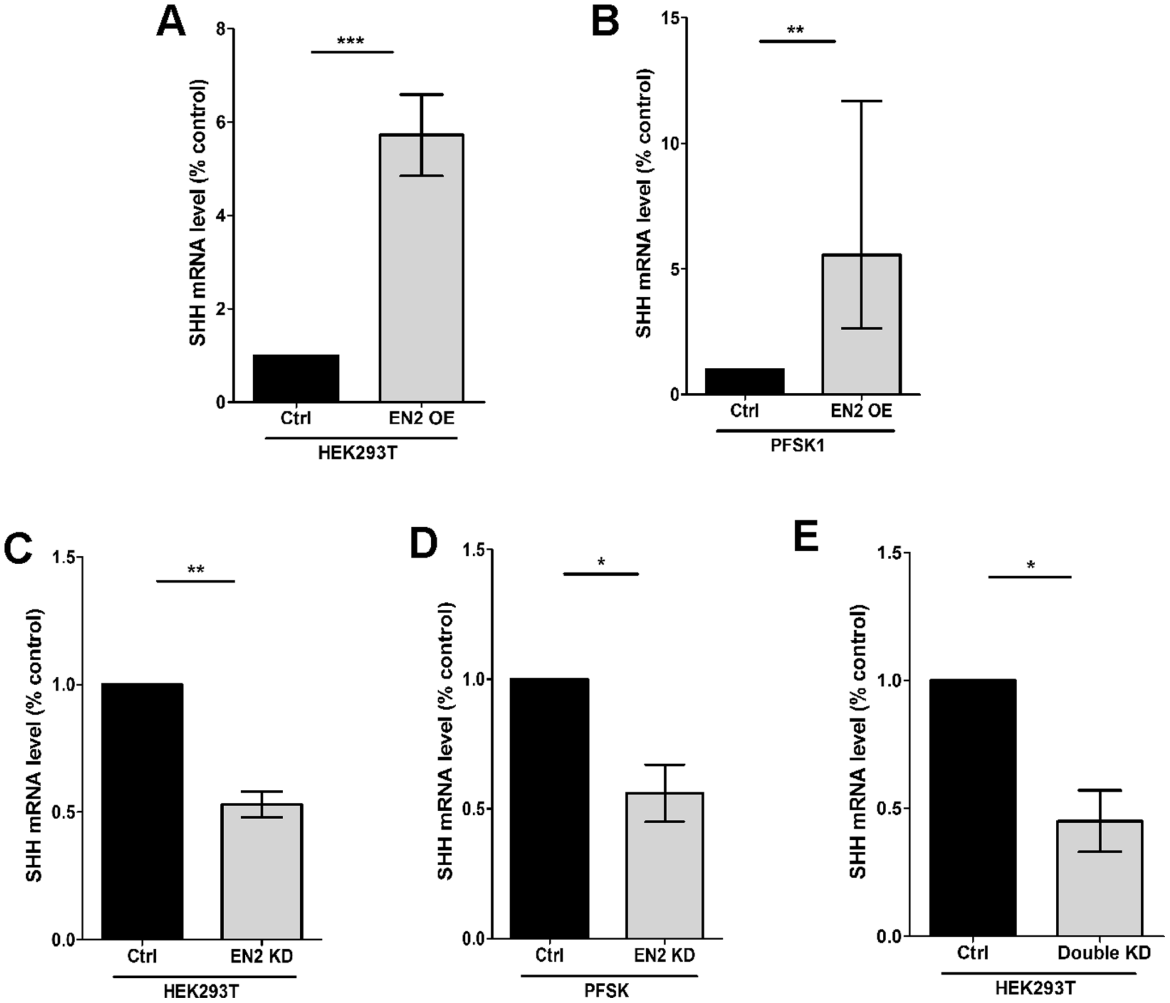
*SHH* expression is regulated by *EN2* levels. To investigate if *EN2* regulates flanking gene expression, over-expression and knock-down analyses were performed. (A, B) For over-expression analysis human *EN2* cDNA (EN2) or empty pCMV-Tag3B vectors (Ctrl) were transfected transiently into HEK293T (A) and PFSK1 (B) cells. SHH is expressed in both cell lines. mRNA levels were measured by Taqman qRT-PCR and normalized to GAPDH. Relative SHH mRNA levels are presented as fold difference of *EN2* over-expression versus control condition. qRTPCR was performed in triplicate and average ΔCt values were used for statistical analyses. N = 8–9, (C, D) *EN2* knock-down (KD) was achieved by transfecting shRNAmir constructs (Open Biosystems) into HEK293T cells (C) and PFSK1 (D) cells. A nonsilencing construct was used as a control (Ctrl). SHH mRNA levels were measured as described above. N = 3–6. (E) Both CUX1 and NFIB bind the *EN2* A-C haplotype and mediate its transcriptional activator function. To investigate the effect of CUX1 and NFIB on the flanking gene levels, stable double knock-downs (KD) were established in HEK293T cells and analyzed. A non-silencing control cell line (Ctrl) was also generated. mRNA levels for each gene were measured using Taqman qRT-PCR. N = 3. In sum *SHH* expression consistently mirrors *EN2* levels in all five experiments. Student T-test, two-tailed, paired, *P<.05, **P<.01, ***P<.001.

To investigate if increased levels of *EN2* could contribute to the altered expression of these flanking genes, *EN2* was over-expressed in non-neuronal (HEK293T) and neuronal (PFSK-1) cell lines, Flanking mRNA levels were measured by Taqman qRT-PCR, normalized to *GAPDH*, and compared to cells transfected with a non-silencing or empty vector. For *EN2* over-expression, average *SHH* mRNA levels are increased 5.72 fold in HEK293T cells and 5.5 fold in PFSK-1 cells (Fig 4a, b). We then examined the effect of *EN2* knock-down on flanking gene expression in both cell lines. 72% and 78% knock-down of *EN2* mRNA was observed in HEK293T and PFSK-1 cells respectivelly. Average *SHH* levels are significantly decreased by ∼45% in both HEK293T and PFSK-1 cells (Fig 4c, d).

Next we examined the effect of stable CUX1 and NFIB single and double knock-downs generated in HEK293T cells. CUX1 and NFIB bind the ASD-associated A-C haplotype and mediate its activator function. Previous analysis demonstrated that endogenous *EN2* expression is decreased significantly by 60% only in the double knock-downs. The single knock-downs had no effect on *EN2* levels [9]. In the double knock-down, average *SHH* levels are significantly decreased (Fig 4e).

**Figure 5:**
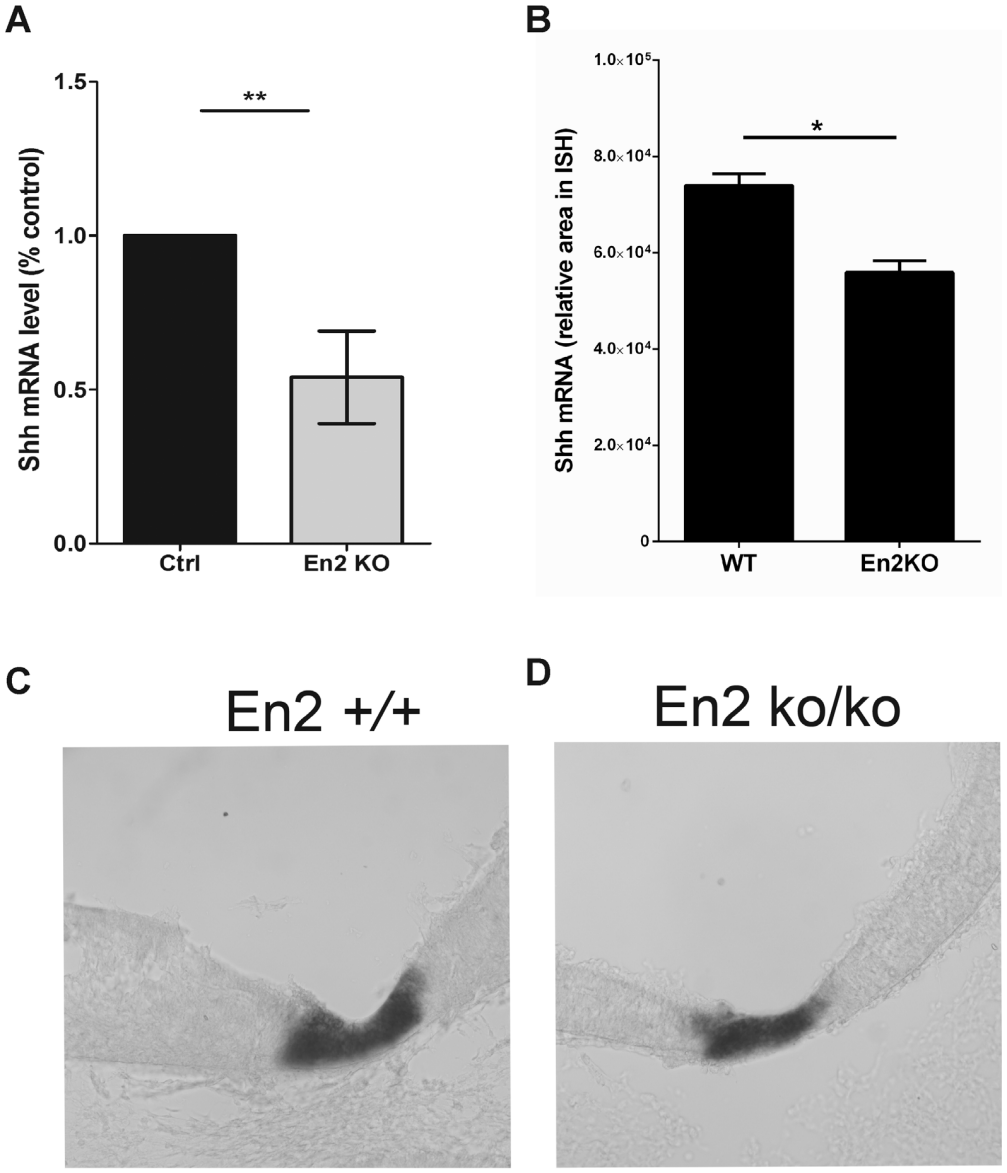
*Shh* levels are decreased in the ventral mid-hindbrain junction of *En2^ko/ko^*. A) Ventral E10.5 mid-hind junction was dissected from *En2* knock-out (KO) embryos and wild-type (WT) littermates. *Shh* mRNA levels were measured by SYBR Green qRT-PCR and reduced expression is observed in the *En2^ko/ko^*. N = 11 Student T-test, two-tailed, paired, **P<.01. B-D) *Shh* ISHs were performed at the same age and a reduced expression area was observed in the KO compared to WT. N = 5 Student T-test, two-tailed, paired, *P<.05.

Finally we investigated the effect of the *En2* knock-out on flanking gene expression. Dopaminergic, noradrenergic and serotonergic neurons are generated from the ventral mid-hindbrain at E10.5. Previous studies indicate that Shh signaling helps coordinate these developmental events [25], [37], [38]. Given the relevance to ASD etiology, mRNA levels were quantitated in micro-dissected mid-hindbrain junction from the *En2* knock-out and wild type littermates. Average *Shh* levels are significantly decreased in the knock-out (Fig 5a). We then examined the spatial expression pattern of *Shh* by ISH. *Shh* is expressed in a ventral domain in the embryonic mid-hindbrain. In the *En2^ko/ko^*, the *Shh* expression domain is reduced (Fig 5c, d). To quantify this difference the entire mid-hindbrain was sectioned, all sections were then subjected to ISH and the average expression domain area was measured. A 24% decrease was observed which is consistent with the reduced mRNA levels (Fig 5a, b)

Transcript levels for some of the flanking genes are also increased or decreased in the above experiments but unlike *SHH* they are never affected in a consistent manner (Figure S4 in File S1). Thus only SHH is consistently positively correlated with EN2 - with over-expression leading to increased *SHH* levels while knock-down/knock-out results in decreased *SHH* levels. In sum all of the above experiments indicate that *SHH* and *EN2* are co-regulated, and suggest that increased levels of *EN2* in the post-mortem samples likely contribute to elevated levels of *SHH*.

## Discussion

The present study demonstrates that *EN2* levels are elevated in affected A-C/G-T individuals, providing additional evidence that *EN2* is an ASD susceptibility gene. These data are consistent with our *in vitro* molecular and biochemical data indicating that the A-C haplotype functions as a transcriptional activator to increase reporter activity and endogenous *EN2* levels. Another recent post-mortem study also demonstrates increased *EN2* levels in cerebellar post-mortem samples but did not correlate expression with the ASD-associated haplotype [10]. Together, these results support the model that increased *EN2* levels contribute to ASD pathogenesis.

Our *EN2* post-mortem analysis demonstrates an interaction between affection status and *rs1861972-rs1861973* genotype. This result indicates that the *rs1861972*-*rs1861973* genotype functions differently depending upon the affection status. In the affected group, individuals with an A-C/G-T genotype express *EN2* at the highest levels. However, in the control group, G-T/G-T individuals display the highest *EN2* levels. These data suggest that other ASD genetic, epigenetic, environmental or treatment factors work in concert with the *rs1861972*-*rs1861973* haplotype to affect *EN2* levels. This finding is consistent with ASD being an epistatic, multi-factorial disorder. The identification of these other ASD factors will aid our understanding of how *rs1861972*-*rs1861973* function is regulated.

Interestingly, affected A-C/G-T individuals display higher *EN2* levels than the affected A-C/A-C group. Given the A-C haplotype functions as a transcriptional activator in cell culture analysis, the simplest model is A-C/A-C individuals should exhibit the highest levels of *EN2* levels. However previous in vitro molecular genetic analysis used standard over-expression and knock-down approaches that never examined the allelic dosage effect of *rs1861972*-*rs1861973* genotype. Possible explanations for why the A-C/A-C genotype did not result in higher EN2 levels include the following. One, autism-specific environmental factors diminish the effect of the A-C haplotype, reducing levels more in A-C/A-C than A-C/G-T or G-T/G-T individuals. For example, prescription drug use and associated comorbid disorders like epilepsy in the autism co-hort could affect *EN2* levels through the A-C haplotype. Some information on medication, comorbidity, and severity of symptoms is available for a subset of affected individuals. Unfortunately the number of individuals with relevant information was not sufficient to warrant a formal statistical test. In addition other unrecorded variables specific to the A-C/A-C autism group likely exist, and these could impact *EN2* levels. Two, other ASD risk factors specifically interact with the A-C/G-T genotype to elevate *EN2* levels. Three, because ASD is a heterogeneous disorder, increased *EN2* levels might only be observed in a subset of affected A-C/A-C individuals, which might be detected if the number of samples was increased substantially. Four, it is possible that the A-C and G-T haplotypes function in trans to create a unique cis-regulatory element. Transvection is a phenomenon whereby cis-regulatory elements function in trans to control gene expression. Reports indicate that transvection regulates gene expression in both humans and Drosophila, suggesting this mechanism is possible [39], [40]

Nevertheless, our post-mortem and molecular analysis is consistent with increased *EN2* levels contributing to ASD pathogenesis. Animal studies indicate that En2 regulates several important aspects of brain development relevant to ASD including connectivity, E/I balance, as well as serotonin and norepinephrine system development [15], [16], [24]-[26], [41], [42]. Over-expression and mis-expression studies in animal models have demonstrated that levels and proper spatial-temporal expression of *En2* is necessary for normal brain development [11], [13]–[15], [43]–[45]. Thus altered levels or mis-expression at a critical time points could affect any number of developmental processes relevant to ASD.

Levels for some of the flanking genes were also significantly increased or decreased in the post-mortem samples (Table 2). *SHH* levels were increased in affected individuals as well as individuals with an A-C/G-T genotype. *INSIG1* levels were decreased in affected individuals, while *CNPY1* was increased in G-T/G-T individuals (Table 2). None of these genes displayed an interaction between genotype and affection status, indicating that ASD only affects *rs1861972*-*rs1861973* regulation of *EN2*. Finally *RBM33* and *HTR5A* did not display any difference in expression.

Because *EN2*, *SHH*, *CNPY1* and *INSIG1* are co-expressed during development and regulate similar developmental processes, we investigated whether elevated levels of *EN2* may contribute to altered flanking gene expression. *SHH* levels were affected in all analyses. When *EN2* was over-expressed, *SHH* levels were also increased as observed in the post-mortem samples. When *EN2* levels were decreased by knock-down, the *CUX1-NFIB* double knock-down, or in the *En2* knock-out, *SHH* levels were also decreased. These results are consistent with *EN2* regulating *SHH* expression, which is supported by research in Drosophila [46]-[48]. Thus one likely down-stream effect of increased *EN2* levels is elevated SHH.

While EN2 regulation of *SHH* likely contributes to increased levels, other factors may also play a role. *SHH* is increased in all affected post-mortem samples regardless of genotype so other ASD risk factors also likely contribute to the increased expression. *SHH* levels are also increased in A-C/G-T individuals, suggesting that the *rs1861972*-*rs1861973* haplotype may regulate *SHH* at a distance. Consistent with this possibility the *CUX1-NFIB* double knock-down displays reduced *SHH* levels while the single CUX1 and NFIB knock-downs had no effect on *SHH* levels. It is well established that enhancers can function hundreds of kilobases away from transcriptional start sites. Interestingly, a classic example of long-range transcriptional regulation is *Shh* where spatially restricted expression in the developing limb, notochord, and floor plate is controlled by distant enhancers [49]-[51]. The cis-regulatory elements important for *Shh* cerebellar expression have not been identified.

Thus EN2 regulation, other ASD risk factors, and the *rs1861972*-*rs1861973* haplotype all likely contribute to increased *SHH* levels. Shh functions as a morphogen during development to coordinate numerous developmental processes including proliferation, neuronal specification, connectivity and synaptogenesis [52], [53]. Shh also plays an essential role in the patterning of the mid-hindbrain to generate ventral dopaminergic, noradrenergic and serotonergic neurons [24], [25], [54], [55]. These neurons then innervate the vast majority of the brain and control behaviors relevant to ASD such as attention, sleep-wake states and anxiety/mood. We observed diminished levels of Shh in the E10.5 *En2* knockout consistent with a possible effect on ventral neurotransmitter development. SHH is also potent mitogen [52]. While many studies point to synaptic defects in autism, macrocephaly and accelerated brain growth have also been consistently reported [56], [57]. Elevated levels of SHH during brain development could contribute to this phenotype. Thus altered SHH levels may contribute to some of the neurodevelopmental phenotypes observed in individuals with ASD.

Finally, *CNPY1* and *INSIG1* levels are also affected in the post-mortem samples but unlike SHH these post-mortem effects do not seem to be due to increased *EN2* levels but instead other ASD factors likely play a more significant role.

In summary, our studies demonstrate that *EN2*, *SHH*, *CNPY1* and *INSIG1* levels are significantly altered in our post-mortem analysis. Increased *EN2* levels are consistent with our previous molecular genetic data for the ASD-associated A-C haplotype. The altered levels of *SHH* is likely to be due to a combination of other ASD-trans acting factors, long range cis-regulatory effects of the *rs1861972*-*rs1861973* haplotype, and regulation by EN2. The *CNPY1* and *INSIG1* effects are more likely due to other ASD risk factors. All four genes function in similar cell biological pathways relevant to ASD, and alterations in their levels could perturb CNS development and contribute to ASD pathogenesis.

## Materials and Methods

All human post-mortem analysis and mouse studies were approved by the University of Medicine and Dentistry of New Jersey (UMDNJ) New Brunswick/Piscataway Institutional Review Board (IRB) and UMDNJ-Robert Wood Johnson Medical School (RWJMS) Institutional Animal Care and Use Committee (IACUC) (I12-013-3) committees. Written consent was obtained from both committees prior to initiating the analysis. All human post-mortem samples were obtained from NICHD Brain and Tissue Bank for Developmental Disorders and the Harvard Brain Tissue Resource Center. All samples are de-identified with no identifiable medical or personal information.

### Post-mortem analysis

We obtained 90 frozen postmortem cerebellar tissue samples from 35 individuals with autism and 55 controls with no obvious diagnosable psychiatric disorder. Frozen cerebellar tissue was homogenized for isolation of RNA and DNA, and each post-mortem sample was genotyped for *rs1861972-rs1861973*. RNA integrity was assessed, cDNA was generated from 78 samples with RIN values greater than 3, and the QRTPCR was performed. See File S1 for details.

### Post-mortem statistical analysis

Statistical analysis was performed in collaboration with biostatistician Yong Lin Ph.D. All results are adjusted for significant covariates. Only *EN2* levels displayed an interaction between genotype and affection status so comparisons were made considering both variables together. For all the other genes, no interaction was observed so comparisons were made for each variable (affection or genotype) separately. Since variations were not the same between the various genotype and affection groups, analysis of covariance with heterogeneous variance model was used to test if affection status or genotype is correlated with significant changes in mRNA levels. The response variable is the average ΔCt, which was generated by performing qRTPCR in triplicate for each transcript in every post-mortem sample. The full model for analysis of covariance includes 10 variables: affection, genotype, sex, and interaction between affection, genotype and sex (affection*genotype, affection*sex, genotype*sex, and affection*genotype*sex), as well as age, post-mortem interval (PMI), and RIN. The Type 3 tests of fixed effects were performed, and insignificant covariates were removed one by one to obtain the final model, which includes only significant covariates. Pair-wise comparisons were conducted based on the final model so any difference in expression levels was adjusted for significant covariates. Since all analysis assumed normality, these assumptions were verified for the final model. Tests for normality of residuals were conducted, and QQ-plots as well as the residuals plots were generated for the final model. Based on these analyses, three potential outliers for *INSIG1* levels (AN16641 and AN00764 - affected G-T/G-T; UMB4899- affected A-C/G-T), one for *CNPY1* (AN16641- affected G-T/G-T), and one for *CUX1* (AN16641- affected G-T/G-T) were detected. For these three genes, sensitivity analysis was performed by removing outliers. Pair-wise comparisons were conducted based on this final model and any difference in expression levels was adjusted for significant covariates. Differences in gene expression levels were estimated for each comparison by calculating least squared mean of ΔΔCt values. Standard error was calculated for each least squared mean and significance was assessed using the Type 3 tests of fixed effects for *EN2* and all flanking genes. Tukey's adjustment was used for all pair-wise comparisons. Fold increase and decrease for each pair was calculated using the formula 2^-least squared mean^. A significance level of 5% was used for all the tests.

### Over-expression, knock-down analysis and knockout analysis

Standard over-expression and knock-down analysis was performed. For the *En2* knock-out analysis, the *En2^tm1Alj^* knockout allele was maintained on a 12∶12 light:dark cycle. Heterozygous matings were performed, impregnated females were identified and sacrificed. The mid-hindbrain junction was dissected underneath a Nikon stereo microscope in 1xPBS using forebrain-midbrain junction and rhombomere 4 as landmarks. The tissue was flash frozen, total RNA was isolated as described above and yolk sac DNA was used to determine genotype. For the over-expression, knock-down and knock-out analysis, flanking mRNA levels were measured by standard SYBR green QRTPCR. See File S1 for details.

## Supporting Information

File S1 **Supporting figures and tables.**
(PDF)
