# Supplementary material for: Autism Associated Gene, ENGRAILED2, and Flanking Gene Levels Are Altered in Post-Mortem Cerebellum
Source: PLoS One. 2014 Feb 10;9(2):e87208. doi: 10.1371/journal.pone.0087208 (PMC3919719; doi:10.1371/journal.pone.0087208)
Supplement: File S1 — Supporting figures and tables. (PDF) [file pone.0087208.s001.pdf]

## SUPPLEMENTAL METHODS

**Post-mortem samples.** 47 samples (13 autism, 35 control) were obtained from NICHD Brain and Tissue Bank for Developmental Disorders, and 43 samples (22 autism, 21 control) are from the Harvard Brain Tissue Resource Center. Tissue was excised from cerebellar vermis (31 samples), cerebellar hemisphere (16 samples), anterior cerebellum (30 samples), cerebellar lobule 6 (12 samples), and unspecified region of cerebellum (1 sample), and kept frozen. Demographic information for each sample is listed in the Supplemental Table 1. 29 autism and 49 control samples were included for statistical analysis.

**RNA and DNA isolation from cerebellar tissue.** Frozen tissue blocks were soaked in 10X volume of RNAlater®-ICE solution (Ambion) and kept at -80°C over night. After removing RNAlater®-ICE solution, 6X volume of cell disruption buffer (Ambion) containing RNasin® Ribonuclease Inhibitor (Promega) and Complete Protease Inhibitor Cocktail tablet (Roche) was added to the tissue. Tissue was homogenized using a hand-held homogenizer (Power Gen 35, Fisher Scientific) for ~30sec. Total RNA of >~200 bases in size was isolated from 350µl of the homogenate using *mir*Vana™PARIS™ kit (Ambion) following manufacturer's instruction. RNA integrity was assessed by following three methods. First, RNA concentration was measured using Biomate3 spectrophotemeter (Thermo Scientific). To examine the quality of RNA, 3µl of each sample was separated on 1% agarose gel in RNase-free condition. RNA Integrity Number (RIN) was then measured using 100-200ng of sample and RNA 6000 Nano kit on Bioanalyzer (Agilent) following manufacturer's instructions. 11 samples displayed RIN<3 and were removed from the analysis.

To isolate genomic DNA, 175µl of homogenate was treated with 0.5µg/µl ProteinaseK (Promega) in buffer containing 60mM Tris, pH8.0, 100mM EDTA, and 0.5% SDS at 55°C over night. Phenol:chloroform:isoamylalcohol (25:24:1) solution was then mixed to the digest. The mixture was centrifuged at14,000 rpm for 10min at 4°C and the upper aqueous layer was collected. The same volume of chloroform was then added and the same procedure was repeated. Collected upper layer was mixed with 1/10 volume of 3M sodium acetate, pH5.2 and one volume of 95% ethanol to precipitate genomic DNA. Precipitated DNA was washed with 70% EtOH and the air-dried pellet was dissolved in 10mM Tris, pH8.0 and 1mM EDTA.

**Genotyping *rs1861972* and *rs1861973*.** Each post-mortem sample was genotyped for *rs1861972* and *rs1861973* using Luminex® technology. Duplex PCR was performed to amplify the regions encompassing *rs1861972* and *rs1861973* using 80ng of genomic DNA, 0.17µM each of forward and reverse primers, 0.2mM of dNTP, 2.5mM of $MgCl_2$, and 0.13µl of AmpliTaq Gold® DNA polymerase (Applied Biosystems) in total of 20µl. Primer sequences are as follows: *rs1861972* forward CCTGCCTATGGGTTGCTTTA; *rs1861972* reverse GGTGATAAGAGGCTCAACCC; *rs1861973* forward CCCTTTCCCCATGGATAGC; *rs1861973* reverse CCAGACATGGGACTGCTTC. PCR cycling conditions are as follows: one cycle at 94°C for 10 min, 40 cycles at 94°C for 40 sec, 60°C for 30 sec, 60°C to 65°C for 1min, and 72°C for 2min, one cycle at 72°C for 10 min. Two microliter of the PCR product was used for ligation detection reaction with 0.01µM each of common primers (*rs1861972* common TGGCCTTGCCCCCTCTATCTTTAAACTACAAATCTAAC; *rs1861973* common CCAAAACCTGGGGCAGGCTATCTTTAAACTACAAATCTAAC) and allele specific primers for each polymorphism (*rs1861972*-A

AATCCTTTTTACTCAATTCAATCACACCACTCCCTGCCAA; *rs1861972*-G CAATTAACTACATACAATACATACCACCACTCCCTGCCAG; *rs1861973*-C CTTTTCAAATCAATACTCAACTTTCCTTACAGCGACCCTGC; *rs1861973*-T CTTTAATCCTTTATCACTTTATCACCTTACAGCGACCCTGT), 0.05µl of Taq DNA ligase (New England Biolabs) in total of 15µl. Conditions for ligation detection reaction are as follows: one cycle at 95°C for 1 min, 31 cycles at 95°C for 15 sec and 58°C for 2 min. Bead hybridization was performed by adding 50µl of mixture containing 0.8µl each of beads, 0.032µM of universal oligo, and hybridization buffer (3M tetramethylammonium chloride, 0.1% SDS, 50mM Tris-HCl, pH8.0, and 3mM EDTA, pH8.0) to the total of ligation detection reaction. Conditions for bead hybridization was as following: 95°C for 2 min, 70°C to 51°C for 1 min per 1°C decrement, 50°C to 38°C for 2 min per 1°C decrement, and 37°C for 20 min. 0.18µl of SAPE and 5.82µl of hybridization buffer was added to the hybridized mixture and incubated at 37°C for 40 min. Fluorescence was then detected on Luminex® 100™ flow cytometry machine. Fluorescence signal from both alleles was collected for each locus and the signal proportion of one allele over both alleles was plotted for each sample. Genotypes were assigned after clustering three genotype groups (two homozygotes and one heterozygote). Samples with ambiguous assignments were re-genotyped.

**Quantitative Reverse Transcription PCR.** First strand cDNA was synthesized from total RNA and quantified using Taqman® real-time qPCR. 3µg of RNA was treated with 2units of DNaseI (New England Biolabs) in a buffer containing 2.5mM $MgCl_2$, 0.5mM $CaCl_2$, and 10mM Tris-HCl, pH7.6 and RNasin® Ribonuclease Inhibitor (Promega) at 37°C for 30min. DNaseI was subsequently inactivated by heating at 75°C for 10min. First strand cDNA was then generated

using 1μg of DNaseI treated RNA and High-Capacity cDNA Reverse Transcription Kit (Applied Biosystems) following manufacturer's instruction. Quantitative PCR was conducted using one twentieth of total cDNA and Taqman® probe sets for human *EN2* (Hs00171321_m1, fluorescent dye FAM labeled) and *GAPDH* internal control (4326317E, fluorescent dye VIC labeled) on ABI7900HT (Applied Biosystems). *EN2* level was normalized to endogenous *GAPDH* level by subtracting *GAPDH* Ct from *EN2* Ct (ΔCt). Average of normalized Ct (ΔCt) values was obtained from three replicates of qRT-PCR reaction. ΔΔCt method was used to calculate relative changes in expression levels. *HTR5A, INSIG1, CNPY1, RBM33,SHH, CUX1,* and *NFIB* levels were measured in the same manner using probe sets Hs00225153_m1, Hs01650979_m1, Hs01073160_m1, Hs00369593_m1, Hs00179843_m1, Hs00738848_m1, and Hs00232149_m1, respectively, where all probes are labeled with fluorescent dye FAM. After Taqman qRT-PCR one sample failed to amplify and was excluded from the analysis.

**Over-expression, knock-down analysis and knockout analysis.** For the *EN2* over-expression and knockdown analysis in human Embryonic Kidney 293T (HEK293T), the cells were maintained in Dulbecco's Modified Eagle Medium (Invitrogen) containing 10% Fetal Bovine Serum at 37 ºC under 5% $CO_2$. Cells were grown to ~85% confluency before human *EN2* protein coding region cloned into pCMV-Tag3B vector or the empty vector was transfected using Lipofectamine2000 (Invitrogen) following manufacturer's protocol. Cells were collected 24hrs after transfection and total RNA was isolated using TRIzol® reagent (Invitrogen) following manufacturer's protocol.  Transient knock-down of *EN2* was generated as described previously (9). The single and double CUX1 and NFIB knock-downs have been reported previously (9). All cell lines were maintained as described above.

*EN2* overexpression and knockdown was also conducted in PFSK-1 cells, which were maintained in RPMI-1 (Invitrogen), 10% Fetal Bovine Serum and 1x Penicillin/Streptomycin at 37C under 5% CO2. Lentivirus was utilized to stably transduce PFSK-1 with the *EN2* overexpression and knockdown constructs. To generate the virus, a second generation lentivirus system was used that included a lentivector (pWPI-CMV-EN2 for overexpression, pGIPZ hEN2 shRNA for knockdown) and packaging plasmids pMD2G and psPAX2. These constructs were co-transfected into HEK-293T cells, virus was harvested 48 hours post-transfection and concentrated using a Beckman ultracentrifuge for 1.5 hours at 25,000xg. Concentrated virus was used to transduce 400,000 cells in media supplemented with 8ug/ml of polybrene with an MOI>1. Fresh media was added 24 hours post-transduction and cells were collected 24 hours following media change. RNA was extracted using Ribozol (AMRESCO) following manufacturer's protocol and processed for qRT-PCR as previously described.

For over-expression, knock-down and knock-out analysis, flanking transcript levels were measured by Taqman® qRT-PCR as described earlier. The following primers were used: (*Gapdh*: F TGTTCCTACCCCCAATGTGTC, R GGAAGTTGCTGTTGAAGTCGCA; *Shh* F GGATGAGGAAAACACGGGAGCA, R TCATCCCAGCCCTCGGTCACT; *Cnpy1* F CAGCTAGATGGTTTGCCTCCTG, R TATGCTCCGAGGAAGGGAGACA; *Insig1* F GACGAGGTGATAGCCACCAT, R ACTTGTGTGGTTCTCCCAGG) and PCR conditions (95C 1' 1 cycle; 95C 30'', 55C 30", 72C 40" 45 cycles) primer sequence and PCR conditions. ΔΔCt method was used to measure gene expression differences compared to controls. To test for significance two-tailed, paired Student T-test was performed for 3-10 independent experiments using average ΔCt values

ISHs for Shh were performed as described [2]. The 3'UTR was PCR amplified (F CATTCTCATCAACCGGGTGC; R AAGGCAGTACAGAAGACGGC cloned and anti-sense and sense probes were generated. 60 micron transverse sections were generated, ISHs were performed on all sections and then the Shh expression domain was quantified by ImageJ for all sections in stage-matched embryos

## SUPPLEMENTAL TABLES

### Table S1. Demographic information of post-mortem samples.

| Individual ID[a] | Diagnosis | Age | Sex | PMI[b] | RIN[c] | Ethnicity | Cause of Death | *rs1861972* | *rs1861973* |
|---|---|---|---|---|---|---|---|---|---|
| UMB 4671 | Autism - confirmed by ADI-R | 4.5 | F | 13 | 3.2 | African American | Multiple injuries | AA | CC |
| UMB 1638 | Autism - confirmed by ADI-R | 20.8 | F | 50 | 3.8 | English-White | Seizure disorder | AA | CC |
| UMB 4231 | Autism - Suspected | 8.8 | M | 12 | 4.6 | African American | Drowning | AA | CC |
| AN19511 | Autism - confirmed by ADI-R | 8 | M | 22.16 | 4.6 | English-White | Cancer (sarcoma) | AA | CC |
| AN08873 | Autism - confirmed by ADI-R | 5 | M | 25.5 | 4.9 | English-White | Asphyxia due to drowning | AA | CC |
| AN09714 | Autism - confirmed by ADI-R | 60 | M | | 5 | Unknown | Pancreatic Cancer | AA | CC |
| AN11989 | Autism - confirmed by ADI-R | 30 | M | 16.06 | 5.4 | Northern European White | Congestive Heart Failure | AA | CC |
| AN01227 | Autism - Suspected | 82 | M | 24.67 | 5.5 | English-White | Arteriosclerotic Cardiovascular Disease | AA | CC |
| UMB 5176 | Autism - confirmed by ADI-R | 22.5 | M | 18 | 6.4 | African American | Subdural hemorrhage | AA | CC |
| UMB 4721 | Autism - confirmed by ADI-R | 8.8 | M | 16 | 6.5 | African American | Drowning | AA | CC |
| UMB 5027 | Autism - Suspected | 38 | M | 26 | 7 | African American | Obstruction of bowel due to adhesion | AA | CC |
| AN06420 | Autism - confirmed by ADI-R | 39 | M | 13.95 | 7.2 | English-White | Cardiac Tamponade | AA | CC |
| AN13872 | Autism - confirmed by ADI-R | 5 | F | 32.73 | 7.2 | Asian | Drowning | AA | CC |
| AN00493 | Autism - confirmed by ADI-R | 27 | M | 8.3 | 3.2 | English-White | Drowning | AG | CT |
| AN08792 | Autism - confirmed by ADI-R | 30 | M | 20.33 | 3.2 | English-White | Gastrointestinal bleeding | AG | CT |
| AN17777 | Autism - confirmed by ADI-R | 49 | F | 16.33 | 3.6 | Unknown | Cardiopulmonary Arrest | AG | CT |
| AN16115 | Autism - confirmed by ADI-R | 11 | F | 12.88 | 4.6 | English-White | Drowning | AG | CT |
| UMB 4899 | Autism - confirmed by ADI-R | 14.3 | M | 9 | 4.9 | English-White | Drowning | AG | CT |
| AN01570 | Autism - confirmed by ADI-R | 18 | F | 6.75 | 5.2 | English-White | Seizure | AG | CT |

| | | | | | | | | | |
|---|---|---|---|---|---|---|---|---|---|
| AN097 30 | Autism - confirmed by ADI-R | 22 | M | 25 | 5.5 | English-White | Aspiration, seizure | AG | CT |
| UMB 4999 | Autism - Suspected | 20. 8 | M | 14 | 6 | English-White | Cardiac arrthymia | AG | CT |
| AN046 82 | Autism - Suspected | 15 | M | 23. 23 | 6.8 | Unknown | Asphyxia, hanging | AG | CT |
| UMB 5115 | Autism - confirmed by ADI-R | 46. 4 | M | 29 | 6.8 | English-White | Complications of pseudomyxoma peridonci | AG | CT |
| AN081 66 | Autism - confirmed by ADI-R | 29 | M | 43. 25 | 7.8 | Eastern Mediterran ean | Seizure Suspected | AG | CT |
| UMB 5144 | Autism - confirmed by ADI-R | 7.2 | M | 3 | 8.1 | Caucasian | Cancer | AG | CT |
| AN124 57 | Autism - confirmed by ADI-R | 29 | F | 17. 83 | 4.2 | English-White | Seizure Disorder | GG | TT |
| AN166 41 | Autism - confirmed by ADI-R | 9 | M | 27 | 4.6 | English-White | Seizure disorder | GG | TT |
| AN036 32 | Autism - Suspected | 49 | F | 21. 08 | 5.2 | Unknown | Cancer | GG | TT |
| AN007 64 | Autism - confirmed by ADI-R | 20 | M | 23. 66 | 6.5 | English-White | Auto Trauma | GG | TT |
| AN032 17 | Control | 19 | M | 18. 58 | 3.1 | English-White | Pneumonia | AA | CC |
| AN121 37 | Control | 31 | M | 32. 92 | 3.8 | Unknown | Asphyxia | AA | CC |
| UMB 818 | Control | 27. 2 | M | 10 | 4.6 | Caucasian | Multiple injuries | AA | CC |
| UMB 4543 | Control | 28. 7 | M | 13 | 5.1 | Caucasian | Multiple injuries | AA | CC |
| UMB 1499 | Control | 4.5 | F | 21 | 5.3 | Asian | Lymphocytic myocarditis | AA | CC |
| AN106 79 | Control | 41 | F | 14 | 5.5 | Unknown | | AA | CC |
| AN178 68 | Control | 46 | M | 18. 78 | 5.5 | Unknown | Heart Attack | AA | CC |
| AN111 84 | Control | 64 | M | 27. 68 | 5.5 | Unknown | Cardiac Arrest | AA | CC |
| UMB 1860 | Control | 8 | M | 5 | 5.6 | Caucasian | Cardiac arrhythmia | AA | CC |
| UMB 1080 | Control | 16. 5 | M | 21 | 5.6 | African American | Cardiac arrythmia, seizure | AA | CC |
| UMB 1706 | Control | 8.6 | F | 20 | 5.7 | African American | Rejection of cardiac allograft transplantation | AA | CC |
| UMB 5173 | Control | 10. 8 | F | 10 | 6 | Caucasian | Asthma | AA | CC |
| AN025 83 | Control | 68 | M | 16. 58 | 6.6 | Unknown | Ruptured Aortic Aneurysm | AA | CC |

| | | | | | | | | | |
|---|---|---|---|---|---|---|---|---|---|
| UMB 1226 | Control | 23.2 | M | 21 | 6.8 | Caucasian | Drowning | AA | CC |
| AN08677 | Control | 38 | M | 25.47 | 6.8 | Unknown | | AA | CC |
| UMB 1846 | Control | 20.6 | F | 9 | 6.9 | Caucasian | Multiple injuries | AA | CC |
| UMB 3 | Control | 5.4 | M | 17 | 7 | Caucasian | Drowning | AA | CC |
| UMB 4670 | Control | 4.6 | M | 17 | 7 | Caucasian | Commotio cordis | AA | CC |
| UMB 1710 | Control | 26.1 | F | 12 | 7.1 | Caucasian | Cardian temponade | AA | CC |
| UMB 4722 | Control | 14.5 | M | 16 | 7.1 | Caucasian | Multiple injuries | AA | CC |
| UMB 1708 | Control | 8.1 | F | 20 | 7.1 | African American | Compressional asphyxia and multiple injuries | AA | CC |
| UMB 1790 | Control | 13.7 | M | 18 | 7.2 | Caucasian | Multiple injuries | AA | CC |
| UMB 1284 | Control | 3.3 | F | 11 | 7.5 | African American | Drowning | AA | CC |
| UMB 1670 | Control | 13.3 | M | 5 | 7.6 | Caucasian | Asphyxia by hanging | AA | CC |
| UMB 5180 | Control | 1.7 | M | 25 | 7.8 | Caucasian | Drowning | AA | CC |
| UMB 1793 | Control | 11.7 | M | 19 | 8.1 | African American | Drowning | AA | CC |
| AN02456 | Control | 4 | F | 17.02 | 5.1 | Unknown | Acute Bronchopneumonia After Tonsillectomy | AG | CT |
| AN17425 | Control | 16 | M | 26.16 | 3.2 | Unknown | Heart Attack | AG | CT |
| AN12552 | Control | 56 | M | 23.61 | 4.4 | African American | Multiple Injuries | AG | CT |
| AN15566 | Control | 32 | F | 28.92 | 4.9 | Unknown | | AG | CT |
| AN12240 | Control | 51 | M | 4.75 | 5.4 | English-White | Heart Attack | AG | CT |
| UMB 1185 | Control | 4.7 | M | 17 | 5.4 | Caucasian | Drowning | AG | CT |
| UMB 1864 | Control | 2.5 | F | 8 | 5.7 | Caucasian | Laryngitis, and bronchiolitis | AG | CT |
| AN01410 | Control | 41 | M | 27.17 | 5.8 | Unknown | | AG | CT |
| AN15622 | Control | 30 | M | 14.83 | 6.1 | English-White | Asphyxia | AG | CT |
| UMB 1297 | Control | 15.2 | M | 16 | 6.5 | African American | Multiple injuries | AG | CT |

| | | | | | | | | | |
|---|---|---|---|---|---|---|---|---|---|
| UMB 1582 | Control | 42.1 | M | 24 | 6.6 | Caucasian | Cardiac arrythmia | AG | CT |
| UMB 1541 | Control | 20.6 | F | 19 | 6.7 | Caucasian | Head injury | AG | CT |
| UMB 1407 | Control | 9.1 | F | 20 | 6.9 | African American | Asthma | AG | CT |
| AN04432 | Control | 22 | M | 24.3 | 6.9 | English-White | Central Hepatic Laceration | AG | CT |
| AN13295 | Control | 56 | M | 22.12 | 7 | Unknown | | AG | CT |
| UMB 1674 | Control | 8.9 | M | 36 | 7.1 | Caucasian | Hyperthermia and drowning | AG | CT |
| UMB 1136 | Control | 33.99 | F | 19 | 7.3 | Caucasian | Arteriosclerotic cardiovascular disease | AG | CT |
| UMB 4590 | Control | 20.5 | M | 19 | 7.6 | Caucasian | Dialated cardiomyopathy | AG | CT |
| AN10833 | Control | 22 | M | 21.47 | 7.6 | Unknown | | AG | CT |
| UMB 1714 | Control | 12.4 | M | 22 | 4.4 | African American | cardiac arrthymia | GG | TT |
| AN17344 | Control | 47 | M | 23 | 5.3 | English-White | Unknown | GG | TT |
| UMB 4787 | Control | 12.9 | M | 15 | 7.4 | African American | Asthma | GG | TT |
| AN14771 | Control | 30 | M | 23 | 8.2 | English-White | Cardiac arrhythmia | GG | TT |

a- Anonymous ID for each sample
b- Post-mortem interval
c- RNA integrity number

**Table S2. Age and Sex distribution of Autism and control groups.**

| Age group (years) | Autism Male | Control Male | Autism Female | Control Female |
|---|---|---|---|---|
| 0-6 | 2 | 4 | 3 | 3 |
| 7-12 | 6 | 6 | 2 | 4 |
| 13-18 | 2 | 8 | 1 | - |
| 19-35 | 8 | 11 | 2 | 6 |
| 36-50 | 4 | 5 | 2 | 1 |
| 51-65 | 3 | 5 | - | - |
| 66-82 | 1 | 1 | - | - |

**Table S3. Distribution of individuals for affection status and *rs1861972-rs1861973* genotype**[a]

| | Autism | Control |
|---|---|---|
| AC/AC | 13 | 26 |
| AC/GT | 12 | 19 |
| GT/GT | 4 | 4 |
| Sum | 29 | 49 |

[a]-The distribution is shown for 78 individuals included in statistical analysis.

**Table S4. Covariate analysis of *EN2* levels for final model[a]**

| Covariate | P-value[c] |
|---|---|
| Age | **0.0174** |
| PMI | **0.0224** |
| RIN | **<.0001** |
| Affection | 0.1144 |
| Genotype | 0.0677 |
| Affection*Genotype[b] | **0.0006** |

[a]- The full model includes all 10 covariates. Insignificant covariates were removed sequentially to obtain the final model.
[b]- Interaction between affection status and genotype
[c]- Type 3 tests of fixed effects. Probability for each covariate is adjusted after considering effects of all significant covariates. Significant values (5% cut-off) are in bold.

**Table S5. *CUX1* levels comparisons based on genotypes or affection status**

| Comparison | Least squared mean[a] | Standard errors[b] | P-value[c] | Fold change[d] |
|---|---|---|---|---|
| AC/AC vs. AC/GT | -0.1184 | 0.1079 | 0.5188 | 1.0855 |
| AC/AC vs. GT/GT | 0.2630 | 0.2415 | 0.5240 | 0.8334 |
| AC/GT vs. GT/GT | 0.3814 | 0.2504 | 0.2864 | 0.7677 |
| Autism vs. Control | 0.08475 | 0.1258 | 0.5029 | 0.9429 |

[a]- Least squared means were calculated from ΔΔCt values ($\Delta Ct^{autism}$-$\Delta Ct^{control}$ or $\Delta Ct^{1st\ genotype}$-$\Delta Ct^{2nd\ genotype}$) after adjusting for significant covariates

[b]- Standard error for estimated difference

[c]- Tukey-Kramer test was performed considering all the significant covariates. No significant difference was observed (5% cut-off).

[d]- Fold change of each first group versus the second is calculated as $2^{-(\text{least squared mean})}$.

**Table S6. *NFIB* levels comparisons based on genotypes or affection status**

| Comparison | Least squared mean[a] | Standard errors[b] | P-value[c] | Fold change[d] |
|---|---|---|---|---|
| AC/AC vs. AC/GT | -0.1004 | 0.1165 | 0.6661 | 1.0721 |
| AC/AC vs. GT/GT | 0.3379 | 0.2544 | 0.3843 | 0.7912 |
| AC/GT vs. GT/GT | 0.4383 | 0.2579 | 0.2125 | 0.7380 |
| Autism vs. Control | 0.01094 | 0.1277 | 0.9320 | 0.9924 |

[a]- Least squared means were calculated from ΔΔCt values ($\Delta Ct^{autism}$-$\Delta Ct^{control}$ or $\Delta Ct^{1st\ genotype}$-$\Delta Ct^{2nd\ genotype}$) after adjusting for significant covariates

[b]- Standard error for estimated difference

[c]- Tukey-Kramer test was performed considering all the significant covariates. No significant difference was observed (5% cut-off).

[d]- Fold change of each first group versus the second is calculated as $2^{-(least\ squared\ mean)}$.

**Table S7. Covariate analysis of *SHH* levels for final model[a]**

| Covariate | P-value[b] |
|---|---|
| RIN | **0.0009** |
| Affection | **0.0327** |
| Genotype | **0.0391** |

[a]- The full model includes all 10 covariates. Insignificant covariates were removed sequentially to obtain the final model.
[b]- Type 3 tests of fixed effects. Probability for each covariate is adjusted after considering effects of all significant covariates. Significant values (5% cut-off) are in bold.

**Table S8. Covariate analysis of *INSIG1* levels for final model[a]**

| Effect | P-value[b] |
|---|---|
| Age | 0.0564 |
| Sex | **0.0100** |
| PMI | **0.0002** |
| RIN | **<.0001** |
| Affection | **0.0352** |
| Genotype | 0.5873 |

[a]- The full model includes all 10 covariates. Insignificant covariates were removed sequentially to obtain the final model.
[b]- Type 3 tests of fixed effects. Probability for each covariate is adjusted after considering effects of all significant covariates. Significant values (5% cut-off) are in bold.

**Table S9. Covariate analysis of *CNPY1* levels for final model[a]**

| Covariate | P-value[b] |
|---|---|
| RIN | **<.0001** |
| Affection | 0.6627 |
| Genotype | **0.0007** |

[a]- The full model includes all 10 covariates. Insignificant covariates were removed sequentially to obtain the final model.
[b]- Type 3 tests of fixed effects. Probability for each covariate is adjusted after considering effects of all significant covariates. Significant values (5% cut-off) are in bold.

**Table S10. *SHH* levels comparisons based on genotypes or affection status**

| Comparison | Least squared mean[a] | Standard errors[b] | P-value[c] | Fold change[d] |
|---|---|---|---|---|
| AC/AC vs. AC/GT | 0.3735 | 0.1435 | **0.0297** | 0.7719 |
| AC/AC vs. GT/GT | 0.1430 | 0.2936 | 0.8777 | 0.9056 |
| AC/GT vs. GT/GT | -0.2306 | 0.3021 | 0.7266 | 1.1733 |
| Autism vs. Control | -0.3290 | 0.1512 | **0.0327** | 1.2561 |

[a]- Least squared means were calculated from ΔΔCt values ($\Delta Ct^{autism}$-$\Delta Ct^{control}$ or $\Delta Ct^{1st\ genotype}$-$\Delta Ct^{2nd\ genotype}$) after adjusting for significant covariates

[b]- Standard error for estimated difference

[c]- Tukey-Kramer test was performed considering all the significant covariates. Significant values (5% cut-off) are in bold.

[d]- Fold change of each first group versus the second is calculated as $2^{-(least\ squared\ mean)}$.

**Table S11.** ***INSIG1*** **levels comparisons based on genotypes or affection status**

| Comparison | Least squared mean[a] | Standard errors[b] | P-value[c] | Fold change[d] |
|---|---|---|---|---|
| AC/AC vs. AC/GT | -0.05403 | 0.08223 | 0.7890 | 1.0381 |
| AC/AC vs. GT/GT | -0.1703 | 0.1815 | 0.6184 | 1.1253 |
| AC/GT vs. GT/GT | -0.1162 | 0.1799 | 0.7952 | 1.0839 |
| Autism vs. Control | 0.1960 | 0.09114 | **0.0352** | 0.8730 |

[a]- Least squared means were calculated from ΔΔCt values ($\Delta Ct^{autism}$-$\Delta Ct^{control}$ or $\Delta Ct^{1st\ genotype}$-$\Delta Ct^{2nd\ genotype}$) after adjusting for significant covariates

[b]- Standard error for estimated difference

[c]- Tukey-Kramer test was performed considering all the significant covariates. Significant values (5% cut-off) are in bold.

[d]- Fold change of each first group versus the second is calculated as $2^{-(least\ squared\ mean)}$.

**Table S12. *CNPY1* levels comparisons based on genotypes or affection status**

| Comparison | Least squared mean[a] | Standard errors[b] | P-value[c] | Fold change[d] |
|---|---|---|---|---|
| AC/AC vs. AC/GT | -0.06545 | 0.1684 | 0.9202 | 1.0464 |
| AC/AC vs. GT/GT | 0.5877 | 0.1758 | **0.0037** | 0.6654 |
| AC/GT vs. GT/GT | 0.6531 | 0.1781 | **0.0013** | 0.6359 |
| Autism vs. Control | -0.07337 | 0.1675 | 0.6627 | 1.0522 |

[a]- Least squared means were calculated from ΔΔCt values ($\Delta Ct^{autism}$-$\Delta Ct^{control}$ or $\Delta Ct^{1st\ genotype}$-$\Delta Ct^{2nd\ genotype}$) after adjusting for significant covariates

[b]- Standard error for estimated difference

[c]- Tukey-Kramer test was performed considering all the significant covariates. Significant values (5% cut-off) are in bold.

[d]- Fold change of each first group versus the second is calculated as $2^{-(least\ squared\ mean)}$.

SUPPLEMENTAL FIGURES

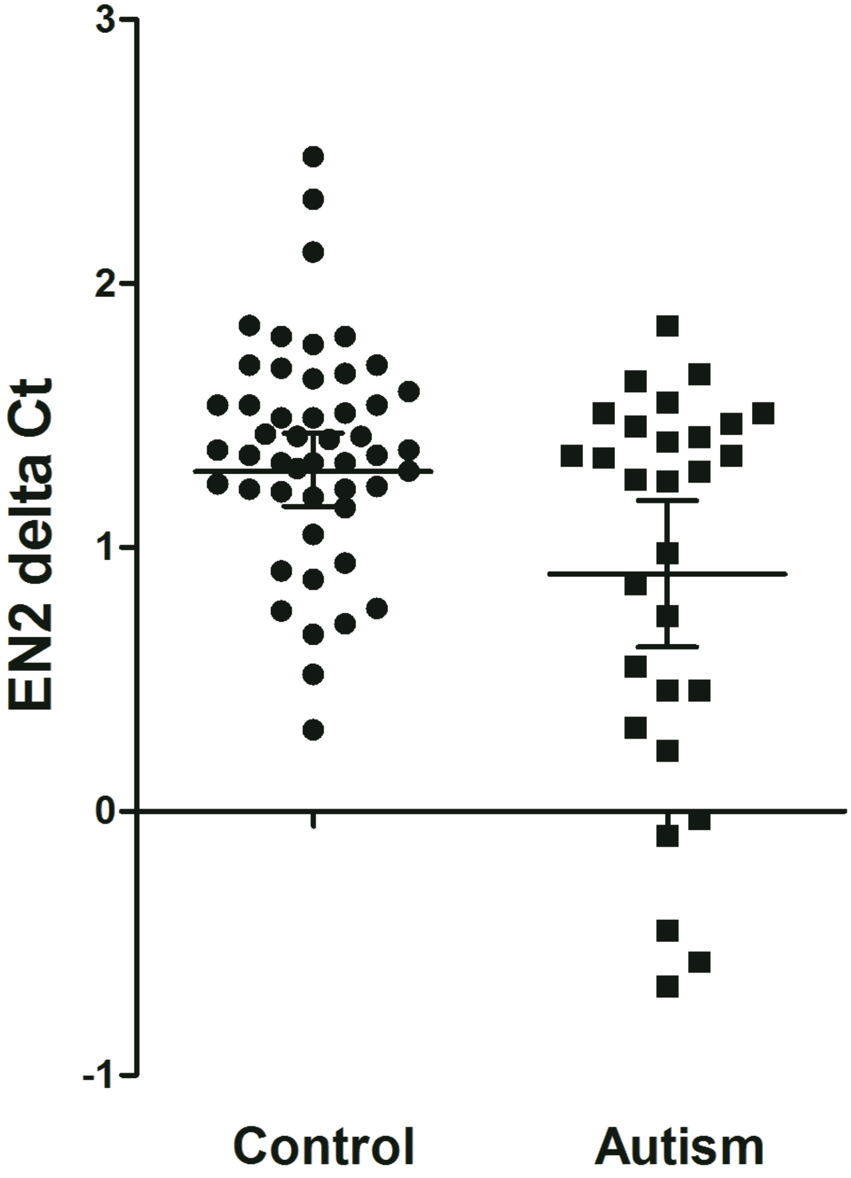


**Figure S1. Distribution of *EN2* levels in control and affected cerebellar post-mortem samples**

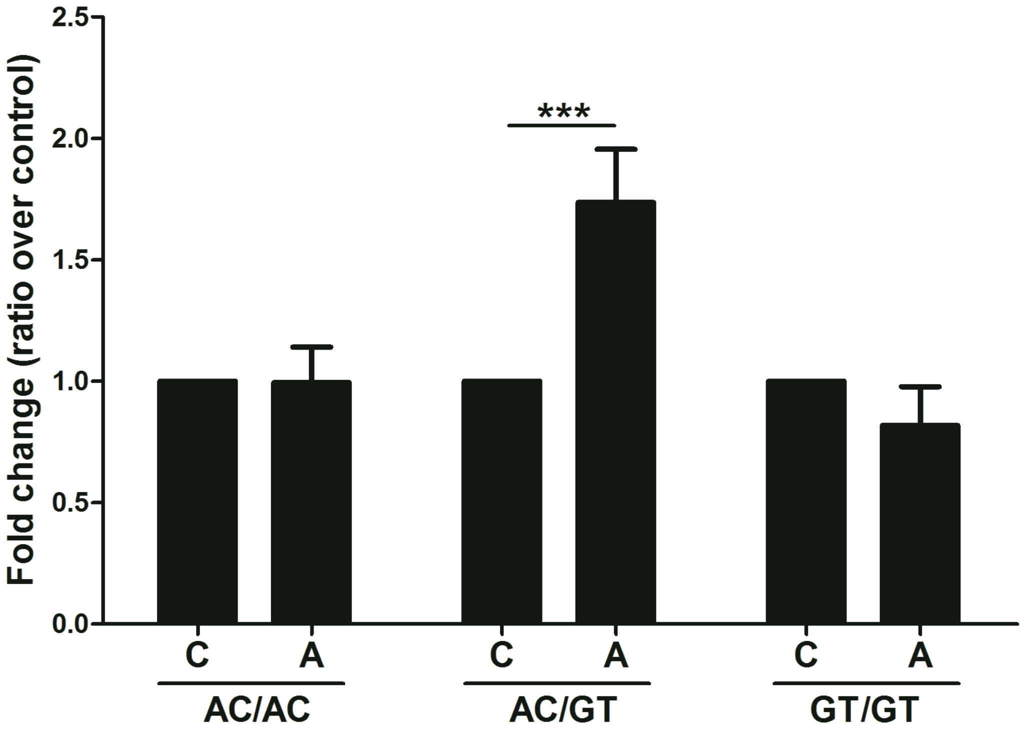


**Figure S2.** ***EN2*** **levels are elevated in affected individuals with an A-C/G-T genotype**

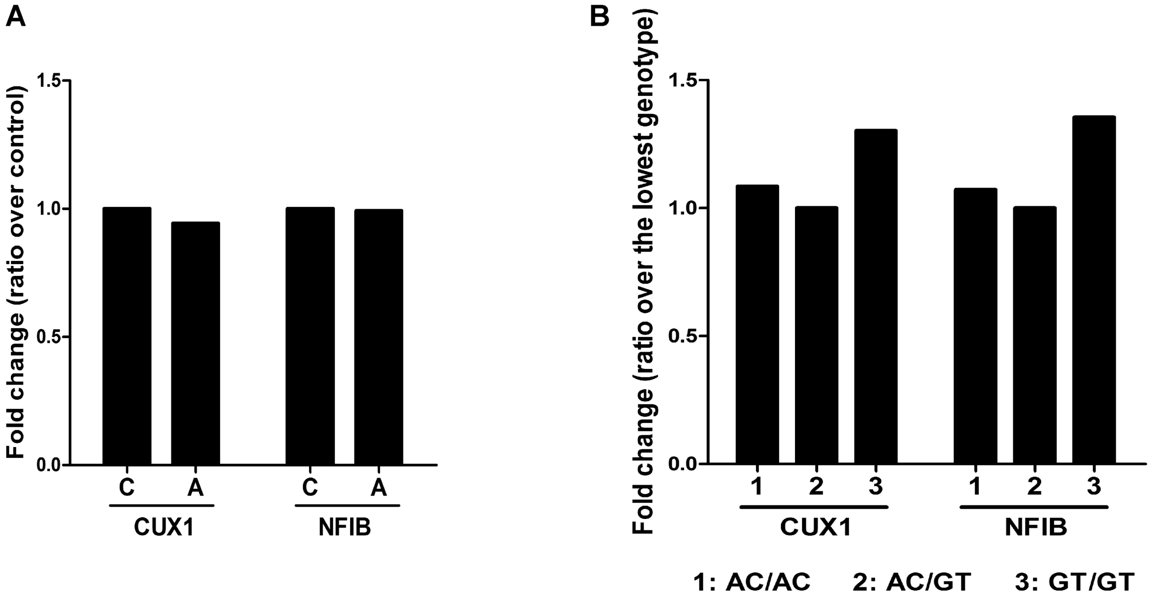


**Figure S3.** ***CUX1*** **and** ***NFIB*** **levels are not correlated with affection status or** ***rs1861972-rs1861973*** **genotype.**

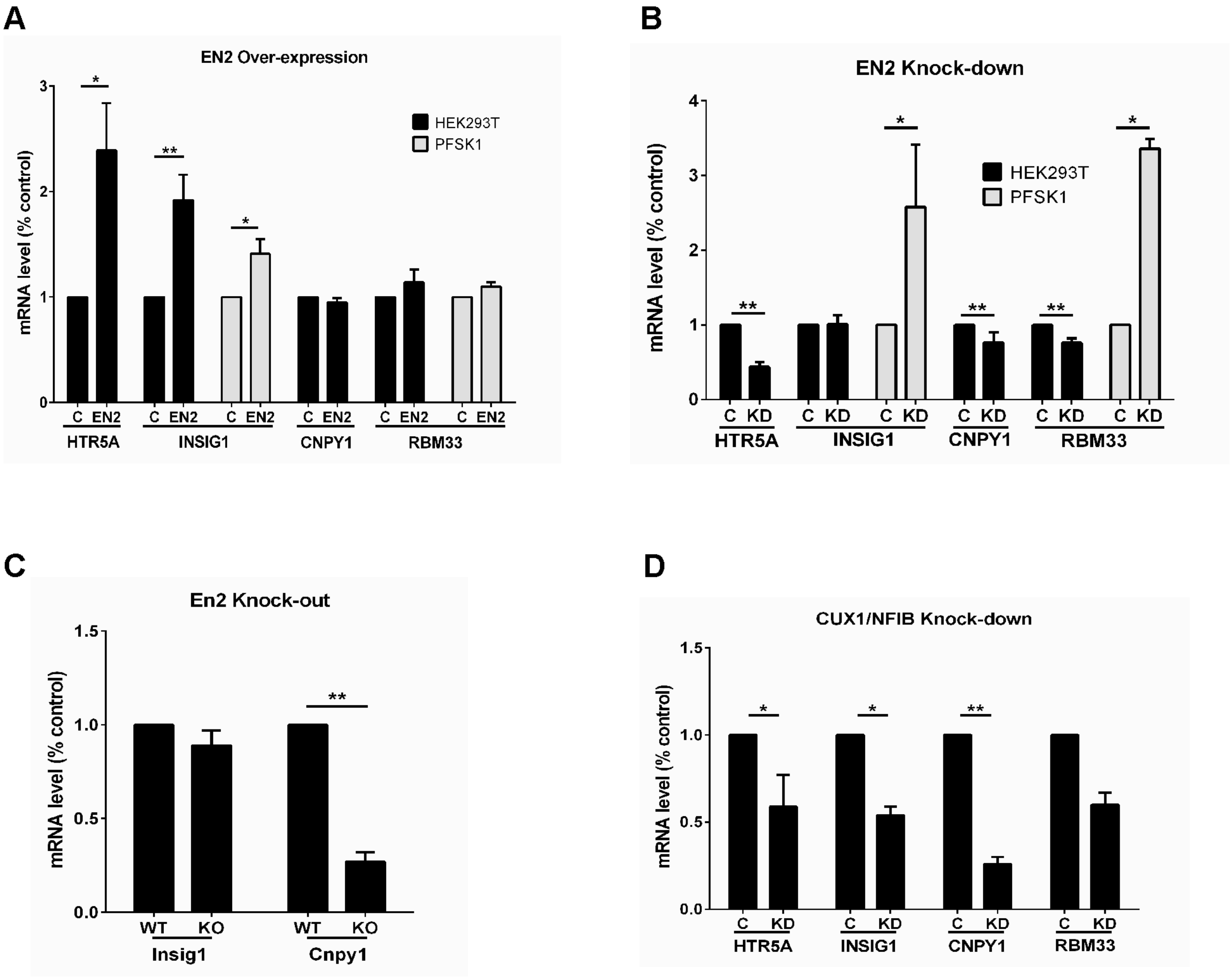


**Figure S4. Over-expression, knock-down and knock-out of *EN2* affects *HTR5A*, *INSIG1*, *CNPY1* and *RBM33* mRNA levels in an inconsistent way**

## SUPPLEMENTAL FIGURE LEGENDS

**Figure S1. Distribution of *EN2* levels in control and affected cerebellar post-mortem samples**

The distribution of normalized *EN2* mRNA levels (DCt) is shown for 29 control and 49 affected cerebellar post-mortem sample. The solid line illustrates the geometric mean and error bars denote 95% confidence intervals. Lower DCt values correspond to higher expression levels

**Figure S2. *EN2* levels are elevated in affected individuals with an A-C/G-T genotype**

Because of the interaction between genotype and affection status, *EN2* levels were compared between genotypes (A-C/A-C, A-C/G-T, G-T/G-T) in control and autism groups separately. In each group *EN2* levels are normalized to 1 for the control group and presented as fold change for the affected individuals. Fold difference was calculated based on ΔΔCt values. Type 3 tests of fixed effects, *P<.05, **P<.01, ***P<.001, ****P<.0001. A – affected, C – control, AC/AC – individuals homozygous for the *rs1861972-rs1861973* A-C haplotype, GT/GT – individuals homozygous for the G-T haplotype, AC/GT – individuals heterozygous for the A-C/G-T haplotype.

**Figure S3. *CUX1* and *NFIB* levels are not correlated with affection status or *rs1861972-rs1861973* genotype.**

mRNA levels of *CUX1* and *NFIB* were measured using Taqman qRT-PCR. No interaction between affection status and genotype was observed so comparisions between autism and control group were

made regardless of genotype (A) and between the three genotypes regardless of affection status (B). (A) Transcript levels are presented as fold change in autism versus control groups. Fold difference was calculated using the formula $2^{-(\text{least squared mean})}$. No significance was observed by Type 3 tests of fixed effects. C – control, A – Autism group. (B) Each gene level is normalized to 1 for the genotype with the lowest quantity (AC/GT for both *CUX1* and *NFIB*). Levels for other genotypes are presented as fold change. Fold difference was calculated using the formula $2^{-(\text{least squared mean})}$. No significance was observed by Type 3 tests of fixed effects.

**Figure S4. Over-expression, knock-down and knock-out of *EN2* affects *HTR5A*, *INSIG1*, *CNPY1* and *RBM33* mRNA levels in an inconsistent way**

To investigate if *EN2* regulates *HTR5A*, *INSIG1*, *CNPY1* and *RBM33* expression, over-expression, knock-down and knock-out analysis were performed. (A) For over-expression analysis human *EN2* cDNA (EN2) or empty pCMV-Tag3B vectors (C) were transfected transiently into HEK293T (black bars) and PFSK1 (gray bars) cells. All flanking genes are expressed in HEK293T cells while only *INSIG1* and *RBM33* are expressed in PFSK1 cells. mRNA levels were measured by Taqman qRT-PCR and normalized to GAPDH. Relative mRNA levels are presented as fold difference of *EN2* over-expression versus control condition. qRTPCR was performed in triplicate and average ΔCt values were used for statistical analyses. N=8-9, (B) *EN2* knock-down (KD) was achieved by transfecting shRNAmir constructs (Open Biosystems) into HEK293T cells (black bars) and PFSK1 (gray bars) cells. A nonsilencing construct was used as a control (C). mRNA levels were measured as described above. N=4-10. (C) Ventral E10.5 mid-hindbrain junction was dissected from $En2^{+/+}$ and $^{ko/ko}$ littermates. Only *Insig1* and *Cnpy1* are expressed at this age, and mRNA levels were measured by SYBR Green qRT-PCR as described. N=11 (D) Both CUX1 and NFIB bind the *EN2* A-C haplotype and mediate its

transcriptional activator function. To investigate the effect of CUX1 and NFIB on the flanking gene levels, stable double knock-downs (KD) were established in HEK293T cells and analyzed. A non-silencing control cell line (C) was also generated. mRNA levels for each gene were measured using Taqman qRT-PCR N=3. Student T-test, two-tailed, paired, *P<.05, **P<.01, ***P<.001.
